# Regionally Metastatic Merkel Cell Carcinoma Associated with Paraneoplastic Anti-*N*-methyl-D-aspartate Receptor Encephalitis

**DOI:** 10.1155/2020/1257587

**Published:** 2020-09-26

**Authors:** Sophia Z. Shalhout, Kevin S. Emerick, Peter M. Sadow, Jenny J. Linnoila, David M. Miller

**Affiliations:** ^1^Division of Hematology/Oncology, Department of Medicine, and Department of Dermatology, Massachusetts General Hospital, Harvard Medical School, Boston, MA 02114, USA; ^2^Department of Otolaryngology, Massachusetts Eye and Ear Infirmary, Harvard Medical School, Boston, MA 02114, USA; ^3^Department of Pathology, Massachusetts General Hospital, Harvard Medical School, Boston, MA 02114, USA; ^4^Department of Neurology, Massachusetts General Hospital, Harvard Medical School, Boston, MA 02114, USA

## Abstract

Merkel cell carcinoma (MCC) is a rare and aggressive cutaneous neuroendocrine cancer with a high risk of recurrence and metastasis. MCC is generally associated with advanced age, fair skin, sun exposure, immunosuppression, and in the majority of cases, the Merkel cell polyomavirus. Neuroendocrine malignancies are associated with a variety of paraneoplastic neurological syndromes (PNS), characterized as autoimmune responses to malignancy-associated expression of neural antigens. Our literature review underscores previous case reports of MCC-associated PNS with voltage-gated calcium channel (VGCC) and anti-Hu (or ANNA-1) autoantibodies. We present the case of a 59-year-old male with regionally metastatic Merkel cell carcinoma complicated by the paraneoplastic manifestation of anti-*N*-methyl-D-aspartate receptor (NMDAR) encephalitis. His primary lower neck subcutaneous MCC and metastasis were initially treated with surgery. Additional recurrent lymph node metastases were successfully treated with definitive intensity-modulated radiation therapy. His PNS improved with rituximab therapy. Although rare, this case highlights that in the setting of seizures and prominent psychiatric symptoms accompanying an MCC diagnosis, evaluation for autoimmune paraneoplastic encephalitis is warranted. Awareness and detection of preexisting PNS are crucial in the era of immune checkpoint inhibitors (ICI) for advanced MCC, where treatment with ICI has the potential to exacerbate preexisting autoimmune PNS and lead to worsened or even lethal neurologic immune-related adverse events (nirAEs).

## 1. Introduction

Paraneoplastic neurological syndromes (PNS) are a heterogenous group of immune-mediated disorders associated with neural autoantibodies directed against antigens expressed by both the tumor and the nervous system. These syndromes may affect any part of the nervous system and are exceptionally rare [1], with a higher incidence in small-cell lung cancer and gynecological tumors [2].

Anti-*N*-methyl-D-aspartate receptor (NMDAR) encephalitis is a recently described PNS. When associated with cancer, NMDAR encephalitis is found to predominantly affect young women with ovarian teratomas [3]. Autoantibody production against the NMDAR leads to internalization of the receptors and profound dysregulation of neurotransmission, with prominent neuropsychiatric manifestations. The onset may begin with a viral-like prodrome, followed by a wide spectrum of clinical features including seizures, memory loss, psychosis, aphasia, and hallucinations [3, 4]. With rare incidence, in case reports, anti-NMDAR encephalitis has been described in association with neuroendocrine differentiated neoplasms of the uterus, pancreas, and liver, as well as small-cell lung carcinomas, typically in older individuals (Table 1).

We report the case of a 59-year-old male who developed anti-NMDAR encephalitis as a paraneoplastic manifestation of Merkel cell carcinoma (MCC). This rare and aggressive cutaneous neuroendocrine cancer has a high risk of recurrence and metastasis, often occurring within the first two years after initial diagnosis [5]. His regionally metastatic MCC was Merkel cell polyomavirus (MCPyV) positive. He underwent surgical-wide local excision with neck lymph node dissection and was successfully treated with definitive intensity-modulated radiation. His anti-NMDAR encephalitis showed slow but steady improvement upon treatment with rituximab. In this report, we highlight the potential complications associated with the evaluation and management of MCC when treatment is initiated for anti-NMDAR encephalitis, since the interpretation of contemporaneous MCPyV oncoprotein antibody serum titers [6] may be affected. Furthermore, the timely identification of an underlying autoimmune PNS is critical when considering immune checkpoint inhibitors (ICI) for the treatment of advanced MCC. Treatment and close monitoring of PNS, including anti-NMDAR encephalitis, before ICI therapy may reduce the possibility of worsened or even lethal neurologic immune-related adverse events [7].

## 2. Case Report

**Figure 1:**
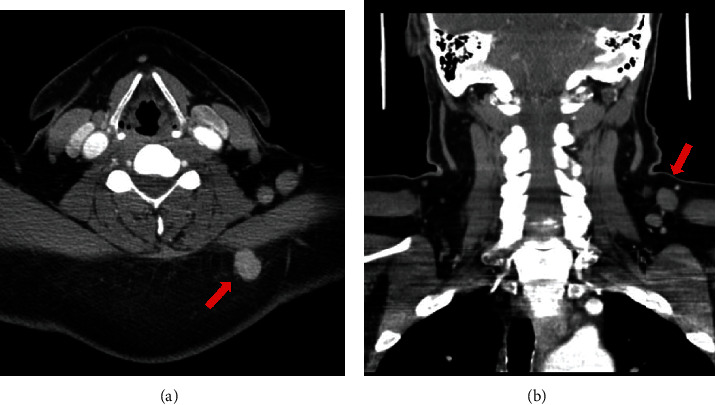
(a, axial view) CT scan with contrast demonstrated a hyperdense rounded mass in the subcutaneous tissue of the left side of the neck, posterior to the trapezius muscle, corresponding to the patient's palpable primary mass (red arrow). (b, coronal view) Several lymph nodes (red arrow) in the posterior triangle anterior to the trapezius muscle ranging in size up to 1.5 cm in diameter were demonstrated on CT scan.

A 59-year-old male with a history of posttraumatic stress disorder and depression presented for evaluation of a palpable mass on the left posterior aspect of his neck that grew over the course of a few months. A computed tomography (CT) scan of the neck with omnipaque contrast demonstrated a hyperdense rounded mass that corresponded to the patient's palpable neck abnormality in the subcutaneous tissue, posterior to the trapezius muscle. Several lymph nodes in the posterior triangle anterior to the trapezius muscle ranging in size up to 1.5 cm in diameter were also appreciated on CT scans (Figure 1).

**Figure 2:**
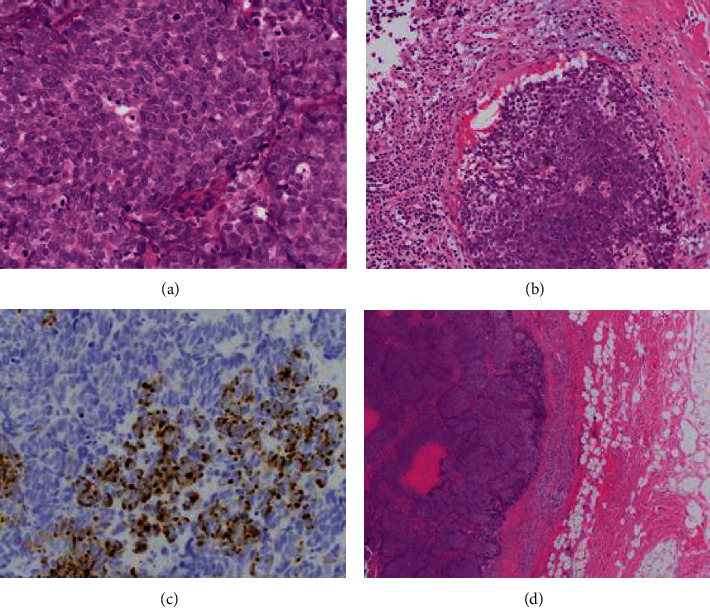
(a) Magnification 400x, hematoxylin and eosin stain of the left neck; Merkel cell carcinoma primary tumor demonstrates numerous mitoses and neuroendocrine nuclei. (b) Magnification 200x, MCC left neck primary highlighting lymphovascular invasion. (c) Magnification 400x, immunohistochemistry demonstrates perinuclear dot-like immunostaining positive for cytokeratin 20. (d) Magnification 40x, MCC primary tumor of the left neck in subcutaneous tissue.

A fine needle aspiration biopsy (FNA) was performed, and a poorly differentiated carcinoma with neuroendocrine features was identified on cytology. An MRI of the brain was unremarkable, and a PET-CT revealed that the nodule in the subcutaneous fat of the left posterior neck was FDG-avid. The left trapezius muscle had six FDG avid nodules in the posterior aspect of the left side of the neck. The patient was taken to the operating room one week later for a wide local excision and a comprehensive level 5 posterior neck lymph node dissection. The immunohistochemical profile of the primary left neck mass demonstrated features consistent with MCC. The tissue was positive for synaptophysin, chromogranin, and cytokeratin 20 in a perinuclear dot-like distribution (Figure 2) as well as CAM5.2 and p16. The cells were negative for keratin 7, p40, TTF-1, PAX 8, and calcitonin. The upper level left neck dissection demonstrated 3 of 8 lymph nodes positive for metastatic MCC, and the lower left neck dissection demonstrated metastatic MCC to 6 of 36 lymph nodes. No extranodal extension was detected. A baseline anti-Merkel cell panel serology test (AMERK, University of Washington Medical Center) was positive for MCPyV oncoprotein antibodies at 7440 standard titer units (STU). The primary tumor was ≤2 cm in diameter, and he presented with clinically and radiologically detected regional lymph node metastasis and therefore was pathologically staged as Stage IIIB (T1pN1b) according to AJCC 8th edition.

Following his excision and lymph node dissection, adjuvant radiotherapy was recommended and planned to manage his MCC. However, prior to the initiation of his adjuvant radiation, the patient presented to the emergency department (ED) of an outside hospital due to seizure activity, with two witnessed seizures in the ED. He was afebrile with the absence of infectious symptoms and displayed expressive aphasia, dyskinesias, altered mental status, and auditory hallucinations. Levetiracetam (750 mg) was initially administered twice daily, and upon discharge, he had another generalized tonic-clonic seizure in the waiting room with a right-sided tongue bite and stool incontinence. He returned to the ED and was administered 1 g of levetiracetam. His aphasia and memory impairment continued, and he was started on empiric acyclovir for herpes simplex virus (HSV) encephalitis coverage, but therapy was discontinued due to acute renal injury. A head CT and brain MRI were unremarkable. A lumbar puncture showed normal chemistries, and his CSF meningoencephalitis panels were negative for any infectious process. He continued to be agitated and confused, with minimal improvement of his aphasia. A repeat brain MRI was unremarkable for abnormalities. The patient's CSF autoimmune encephalopathy panel returned positive for NMDA receptor GluN1 antibodies (1 : 80 titer) and negative for other neural antibodies. His symptoms were attributed to NMDAR encephalitis, likely an autoimmune paraneoplastic syndrome due to his MCC. He received a 5-day course of intravenous immunoglobulin and was started on 60 mg of prednisone per day. His expressive aphasia and neurological exams improved, and he was discharged to a rehab facility on levetiracetam (500 mg twice a day) and divalproex (750 mg twice a day). On the neurologic follow-up at this hospital, the patient still had significant cognitive deficits, particularly in his short-term memory. It was decided to slowly reduce his prednisone dose and to initiate rituximab instead.

**Figure 3:**
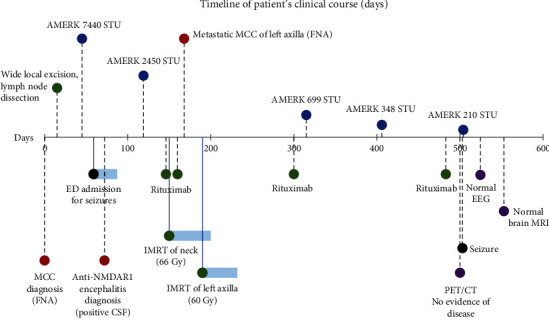
Timeline of patient's clinical course highlighting surgical excision, radiation, and MCPyV antibody panel AMERK results for the treatment and management of his MCC, as well as rituximab infusions for his paraneoplastic autoimmune encephalitis. Day 0 is set to his initial MCC diagnosis. Red: diagnoses; blue: AMERK titers; green: treatment interventions for MCC and encephalitis, including wide local excision, IMRT, and rituximab infusions (1 g); black: seizures; purple: latest follow-up scans and EEG.

A PET-CT three months after his surgical excision demonstrated two FDG-avid nodules along the left levator scapulae muscle. Moderate FDG uptake to his left axillary lymph nodes, consistent with recurrence of his MCC, was also observed. His AMERK test revealed a ~67% decrease in MCPyV oncoprotein serum titers (2450 STU). During this time, he completed two doses of rituximab for his anti-NMDAR encephalitis (Figure 3). He was slowly tapered off of levetiracetam and divalproex. An FNA of his left axillary mass confirmed metastatic MCC, with no evidence of metastatic brain disease demonstrated on MRI. He began intensity-modulated radiation therapy (IMRT) to his neck and left axillae. At this stage, he continued to need assistance with activities of daily living to improve his memory. He successfully completed IMRT and was continued on rituximab, dosed every six months. A few weeks after his latest dose, he experienced a single seizure. He was restarted on levetiracetam. A follow-up routine EEG was normal. His AMERK tests continued to trend downward to 210 STU but remained positive for oncoprotein antibody in serum (AMERK of <74 STU is considered negative) (Figure 3). He continues to be followed every three months for close monitoring, with full body skin exams and surveillance PET-CT scans, which continue to be reassuring for no evidence of recurrence or metastatic MCC disease. His cognitive status has continued to improve on rituximab, with a recent brain MRI revealing no abnormalities and no evidence of intracranial metastatic disease.

## 3. Discussion

Merkel cell carcinoma is an aggressive and rare neuroendocrine skin cancer. This report highlights a patient who was treated with surgical therapy and IMRT for regionally metastatic MCC and achieved complete regional control both clinically and radiologically, one year post definitive radiotherapy. The clinical psychiatric and neurological manifestations in our patient were typical of anti-NMDAR encephalitis, generally characterized by altered mental status, hallucinations, orofacial dyskinesias, seizures, and autonomic instability. The detection of anti-NMDAR antibodies in his CSF makes a paraneoplastic manifestation, with MCC as the inciting malignancy, probable [8]. Large cohort studies have demonstrated that the underlying tumor associated with paraneoplastic anti-NMDAR encephalitis in the majority of cases is an ovarian teratoma, either mature or immature [8, 9]. Nervous tissue components of ovarian teratomas from patients with anti-NMDAR encephalitis were shown to express NMDAR subunits [10]. A few previous cases have described anti-NMDAR encephalitis associated with neuroendocrine neoplasms of the uterus, liver, lung, and pancreas, with 5 reports demonstrating tumors positive for NMDAR subunits (Table 1).

A review of the literature of PNS associated with a diagnosis of MCC highlights case reports of neurologic and prominent psychiatric symptoms associated with VGCC and anti-Hu (or ANNA-1) autoantibodies. Cases describing novel antibodies against nerve fibers or filamentous nervous system structures have also been reported in patients with PNS secondary to MCC (Table 2). We report a rare case of anti-NMDAR encephalitis as a paraneoplastic manifestation due to MCPyV-positive MCC. The treatment of his encephalitis with rituximab complicates the interpretation of the AMERK MCPyV serology tests used to manage virus-positive patients who produce oncoprotein antibodies. An oncoprotein antibody test is typically obtained within 2-3 months of initial evident disease, to establish a baseline in virus-positive MCC patients. After successful treatment, oncoprotein antibodies usually decrease rapidly, falling ~90% one year after successful treatment. However, upon recurrence, metastases, or increased tumor burden, titers increase rapidly [11]. Treating PNS with rituximab in our patient complicates the interpretation of the titer results, and we highlight the need for further studies and exploration of the effects on antibody levels used to track MCC burden in patients treated with anti-CD-20-directed therapies for PNS.

The timely identification and treatment of an underlying autoimmune PNS is critical when considering approved ICI therapies, such as avelumab and pembrolizumab, for the treatment of advanced MCC. Treatment and close monitoring of PNS, including anti-NMDAR encephalitis, before and during ICI therapy, may reduce the potential for worsening or lethal immunotherapy-related neurologic adverse events. PNS are typically associated with poor prognosis, and upon treatment with ICIs, these patients may have worsening of their PNS. Cases of severe and long-term disability as well as death have been reported in patients on anti-PD-1 inhibitors for lung cancer due to exacerbation of preexisting autoimmune PNS, with increased titers of anti-Hu/ANNA-1 and anti-voltage gated potassium channel (VGKC) antibodies [7, 12]. There were also 2 reports of cases of severe disability and death in patients on nivolumab and pembrolizumab for metastatic MCC due to exacerbation of preexisting anti-Hu/ANNA-1 and anti-VGKC autoimmune PNS and nirAEs [13]. Given the rarity of these conditions, awareness of PNS in the setting of prominent neurologic symptoms in association with MCC is of critical importance.

## Figures and Tables

**Table 1:** Reports of neuroendocrine neoplasms associated with anti-NMDA receptor encephalitis.

Reference(s)	Sex/age	Neuroendocrine neoplasm
Lim and Yip [14]	M/65	Hepatic neuroendocrine carcinoma
Bost et al. [9]	F/50	Pancreatic neuroendocrine tumor (NMDAR+ tumor)
Afanasiev et al. [15]	F/51	Pancreatic neuroendocrine tumor (NMDAR+ tumor)
Hara et al. [16]	F/65	Carcinosarcoma with neuroendocrine differentiation of the uterus (NMDAR+ tumor)
Kobayashi et al. [17]	F/44	Large-cell neuroendocrine carcinoma of the uterus (NMDAR+ tumor)
Boangher et al. [18]	F/66	Small-cell lung cancer
Jeraiby et al. [19]	F/62	Small-cell lung cancer (NMDAR+ tumor)
Titulaer et al. [20]	NA	Small-cell lung cancer

**Table 2:** Autoimmune paraneoplastic syndromes associated with Merkel cell carcinoma.

Reference(s)	Symptoms/syndrome	Autoimmune antibodies
Lopez et al. [21]	Painless proximal muscle weakness	Anti-Hu/ANNA-1 antibodies
Balegno et al. [22]	Paraneoplastic cerebellar ataxia	Antibodies against cerebellar nerve fibers
Greenlee et al. [23]	Sensorimotor and autonomic neuropathy, encephalopathy	Anti-Hu/ANNA-1 antibodies
Cher et al. [24]	Paraneoplastic brainstem encephalitis	Antibodies against brain and cerebellum filamentous structures
Eggers et al. [25], Nguyen et al. [26], and Iyer et al. [27]	Lambert-Eaton myasthenic syndrome	Antibodies against VGCCs
Sharobeam et al. [28]	Subacute cerebellar degeneration	Anti-Hu/ANNA-1 antibodies
Zhang et al. [29]	Paraneoplastic cerebellar degeneration	Antibodies against VGCCs
Hocar et al. [30]	Severe necrotizing myopathy	Anti-Hu/ANNA-1 antibodies
Current case	Expressive aphasia, altered mental status, memory loss, seizures/NMDAR encephalitis	Anti-NMDAR antibodies

## Abbreviations

AMERK: Anti-Merkel cell panel antibody titer serology test, University of Washington Medical Center
CSF: Cerebrospinal fluid
ICI: Immune checkpoint inhibitors
FDG: ^18^F-fluorodeoxyglucose
ED: Emergency department
FNA: Fine needle aspiration biopsy
HSV: Herpes simplex virus
IMRT: Intensity modulated radiation therapy
MCC: Merkel cell carcinoma
MCPyV: Merkel cell polyomavirus
MRI: Magnetic resonance imaging
NA: Not available
nirAE: Neurologic immune-related adverse events
NMDAR: *N*-Methyl-D-aspartate receptor
PET-CT: Positron emission tomography-computed tomography
PNS: Paraneoplastic neurological syndrome
STU: Standard titer units
VGCCs: Voltage gated calcium channels
VGKCs: Voltage gated potassium channels.
